# Age-Dependent Effects of Oral Infection with Dengue Virus on *Aedes aegypti* (Diptera: Culicidae) Feeding Behavior, Survival, Oviposition Success and Fecundity

**DOI:** 10.1371/journal.pone.0059933

**Published:** 2013-03-29

**Authors:** Gabriel Sylvestre, Mariana Gandini, Rafael Maciel-de-Freitas

**Affiliations:** 1 Laboratório de Transmissores de Hematozoários, Instituto Oswaldo Cruz - Fiocruz, Rio de Janeiro, Brazil; 2 Laboratório de Imunologia Viral, Instituto Oswaldo Cruz - Fiocruz, Fiocruz, Rio de Janeiro, Brazil; Blood Systems Research Institute, United States of America

## Abstract

**Background:**

*Aedes aegypti* is the main vector of dengue, a disease that is increasing its geographical range as well as incidence rates. Despite its public health importance, the effect of dengue virus (DENV) on some mosquito traits remains unknown. Here, we investigated the impact of DENV-2 infection on the feeding behavior, survival, oviposition success and fecundity of *Ae. aegypti* females.

**Methods/Principal Findings:**

After orally-challenging *Ae. aegypti* females with a DENV-2 strain using a membrane feeder, we monitored the feeding behavior, survival, oviposition success and fecundity throughout the mosquito lifespan. We observed an age-dependent cost of DENV infection on mosquito feeding behavior and fecundity. Infected individuals took more time to ingest blood from anesthetized mice in the 2^nd^ and 3^rd^ weeks post-infection, and also longer overall blood-feeding times in the 3^rd^ week post-infection, when females were around 20 days old. Often, infected *Ae. aegypti* females did not lay eggs and when they were laid, smaller number of eggs were laid compared to uninfected controls. A reduction in the number of eggs laid per female was evident starting on the 3^rd^ week post-infection. DENV-2 negatively affected mosquito lifespan, since overall the longevity of infected females was halved compared to that of the uninfected control group.

**Conclusions:**

The DENV-2 strain tested significantly affected *Ae. aegypti* traits directly correlated with vectorial capacity or mosquito population density, such as feeding behavior, survival, fecundity and oviposition success. Infected mosquitoes spent more time ingesting blood, had reduced lifespan, laid eggs less frequently, and when they did lay eggs, the clutches were smaller than uninfected mosquitoes.

## Introduction

Dengue has become one of the most important vector-borne diseases in the recent decades because of its increasing incidence, high morbidity and economic impact. The World Health Organization (WHO) estimates that 50 million cases of dengue occur per year, with more than 2.5 billion people living at risk of infection in endemic areas. The geographic extension of dengue is primarily confined to the tropical areas of Southeast Asia, the Pacific and the Americas, but regions in Africa and the eastern Mediterranean are increasingly affected [1].

Dengue virus (DENV) belongs to the family *Flaviviridae* and is transmitted between humans through the bite of the mosquito *Aedes aegypti*, its only known natural vector in the Americas. This mosquito species is commonly found in high numbers in urban areas, living close to human dwellings, where females usually blood-feed on humans and 3–4 days later lay eggs in man-made containers [2]–[5]. Thus, reducing or eliminating larval habitat located in and around human habitations has been advocated as an important component of sustainable vector control programs [6]–[9].

It is believed that the intensity of dengue transmission is largely influenced by the parameters governing *Ae. aegypti* vectorial capacity such as the population density of the mosquito, its biting rate, its daily survival rate, its susceptibility to DENV infection and the extrinsic incubation period of the virus [10], [11]. A thorough understanding of DENV epidemiology therefore depends upon a detailed understanding of how these life history traits affect *Ae. aegypti* vectorial competence. Furthermore, little is known about how the mosquito–dengue interaction itself influences these epidemiologically relevant parameters. Like other arboviruses, dengue virus is able to invade the mosquito’s brain, which may modify the mosquito’s physiology, metabolism and behavior, thereby changing vectorial capacity and the pattern of disease transmission [12], [13]. In this context, estimates of the mortality, longevity and biting rate of dengue-infected *Ae. aegypti* still need further characterization [10]–[14]. Most mathematical models assume that there is little or no effect of dengue virus infection on these parameters or if there is, it is not well understood [15]. Recently, some ecological aspects of the interaction between DENV and *Ae. aegypti* have been explored. For instance, it was observed that mosquitoes challenged with DENV had lower survival rates and longevity when compared to those uninfected mosquitoes [16]. Furthermore, DENV had an age-dependent effect on mosquito fecundity, since infected females laid fewer eggs per clutch than uninfected controls in the third and subsequent oviposition cycles [16]. *Ae. aegypti* females infected intra-thoracically with DENV-2 also had an increase of up to 50% in their locomotor activity when compared to uninfected control mosquitoes [17].

Dengue transmission is heavily dependent on mosquito biting behavior. As with other disease vectors, *Ae. aegypti* females are attracted by visual and chemical cues such as carbon dioxide, air movement and heat, as well as body odors from human skin such as lactic acid and ammonia [18]. Once a suitable host is located, the mosquito must obtain blood as quickly as possible to avoid any host defensive behavior [19]. Yet, the potential influence that DENV may play on *Ae. aegypti* biting behavior remains poorly known. For instance, only two studies have considered the biting rate of DENV-infected *Ae. aegypti* and conflicting results were reported [13], [20]. Using mosquitoes from long-established laboratory colonies, Putnam and Scott [20] found no evidence that DENV-2 influences the feeding behavior of the mosquito (i.e. the biting rate). On the other hand, Platt et al. [13] observed that the time required for blood-feeding and the time spent during probing were longer in dengue-infected mosquitoes than in uninfected individuals. Furthermore, the aforementioned papers performed intra-thoracic inoculation to infect mosquitoes, which is an invasive and non-natural mode of transmission that has important consequences on the dynamics of virus-vector interactions [21].

In this study, we compared the effect of oral dengue infection on some life-history traits of *Ae. aegypti* such as the mortality, longevity, age-specific fecundity, oviposition success and biting behavior of two mosquito populations.

## Methods

### Mosquitoes

Two *Ae. aegypti* populations were tested, since previous experiments revealed the effects of DENV-2 infection varied between mosquito populations [16]. The field population was derived from the F1-generation of mosquitoes collected at Fiocruz campus, Rio de Janeiro, using 80 ovitraps filled with hay infusion and distributed in an area of approximately 2.32 hectares, in close proximity to a typical Brazilian slum, which historically has a high incidence of dengue. The laboratory colony used was the Paea strain, which is highly susceptible to oral dengue infection [22]. The colony was initiated with mosquitoes caught in French Polynesia in 1994. Based on the observation of 15–20 generations per year in our laboratory, the Paea strain has been maintained for approximately 250–300 generations [22].

In all assays, larval populations were reared and maintained under identical laboratory conditions. Larvae were reared on yeast extract and raised in plastic basins at 25±3°C. After emergence, adults were allowed to mate and maintained in 45 cm^3^ cages kept at 27±2°C and 75±5% relative humidity and in approximately 12–12 h light-dark photoperiod. They were fed *ad libitum* with cotton soaked in a 10% sucrose solution for up to approximately 36 h before being offered an infectious bloodmeal to the *Ae. aegypti* females.

### Virus

Dengue virus type 2 strain 16681 [23] with 6 passages on C6/36 cells was used for virus stock preparation as described elsewhere [24]. Briefly, *Aedes albopictus* cell clone C6/36 (CRL-1660, ATCC) was maintained at 28°C in Dubelcco’s modified Eagle Medium (Gibco, Life Technologies) with sodium bicarbonate and supplemented with 5% fetal bovine serum (FBS), 1% penicillin-streptomycin-glutamine (Gibco), 0.5% non-essential amino acids (Gibco) and 10% tryptose phosphate (Sigma). C6/36 cell monolayers were infected with DENV-2 and cell culture supernatants were harvested 8 days later. A purified DENV-2 stock was obtained by ultracentrifugation at 100,000 g for 1 h and set to a final volume 20 times smaller than initial [25]. Titration was performed in C6/36 cells using a standard TCID_50_ (50% tissue culture infective dose) assay as described elsewhere [26]. Uninfected flasks were maintained, also purified and used as negative control (MOCK).

### DENV-2 Vector Competence Assay

Before performing the experiments investigating the effect of DENV-2 on *Ae. aegypti* life-history parameters, we carried out controlled preliminary assays to assess the vector competence of the two *Aedes aegypti* populations to our DENV-2 strain. For this, 4–5 days after emergence, 60 inseminated females were placed into small cylindrical plastic cages, with no access to sugar. Approximately 36 h later, they were offered a DENV-2 infectious blood meal, which consisted of one milliliter of viral stock added to 2 ml of washed rabbit erythrocytes. The blood-meal was heated to 37°C and given to the mosquitoes in an artificial membrane feeding apparatus [27]. The mosquitoes were allowed to feed for 25 min on infectious blood that contained a viral titre of 2×10^8^ TCID_50_. Fourteen days post-infection (dpi), the heads of the mosquitoes were examined by indirect immunofluorescence to detect DENV.

Due to the satisfactory susceptibility of *Ae. aegypti* mosquitoes to the DENV-2 strain used in these experiments (data in the Results section), we assumed all mosquitoes from both populations that received the same viral stock were infected.

### Oral Infection of Mosquitoes with DENV-2

For the experiments investigating the effect of DENV-2 on *Ae. aegypti* life-history parameters, for each *Ae. aegypti* population, female mosquitoes were split into two groups, one of which received an infectious blood meal as described above, while the other received a non-infectious blood meal, in which the viral supernatant was replaced with 1 ml of culture medium. The same procedure and apparatus were otherwise used to blood-feed both groups of mosquitoes.

Those females that were visually fully engorged were isolated in labeled cylindrical plastic vials (6.5 cm height, 2.5 cm diameter) containing at the bottom moistened cotton, overlaid with filter paper, as a substrate for oviposition, and closed on the top with mosquito netting.

### *Aedes aegypti* Feeding Behavior

In order to estimate the potential changes in *Ae. aegypti* feeding behavior, we offered an anesthetized mouse to the mosquitoes once a week, for a period of three weeks. At each blood feeding event, (7, 14 and 21 dpi), we measured five different aspects of mosquito blood-feeding.

#### Start time

The period of time elapsed between contact of the vial with the mouse until the mosquito introduced its proboscis into the body of the host. Herein, we aimed to assess the host-seeking behavior and evaluate whether DENV- infected individuals were more efficient in locating the host and initiating the feeding process than uninfected mosquitoes.

#### Number of probings

The number of times a female inserted its proboscis into the host during blood feeding. This parameter is perhaps the most epidemiologically relevant, because an increase in probing may lead to a direct increase in virus transmission.

#### Probing time

The time elapsed between the beginning until the end of probing. This time ended when we observed the abdomen beginning to expand due to blood ingestion. Here, we intended to evaluate whether DENV-infected individuals initiate ingestion of blood more rapidly than uninfected mosquitoes.

#### Ingestion time

The time elapsed from the beginning of abdominal expansion to the end of engorgement, which was characterized by proboscis withdrawal from the host body. Here, we intended to evaluate whether infected individuals would feed to repletion more rapidly than the control group, thus favoring virus transmission by reducing the time vector is exposed to host defensive behavior.

#### Feeding time

The sum of the start, probing and ingestion times. Since feeding time represents the cumulative variation in start, probing and ingestion time, feeding time must not be considered as an independent variable and its variation must be carefully interpreted.

### Mosquito Fecundity and Survival

Three days after each blood meal, the filter papers were removed and checked for eggs, which were counted, and a new filter paper was added as oviposition substrate to the vials. Survival was checked daily at 09∶00 am and 04∶00 pm. When a dead mosquito was observed, it was removed from the plastic tube, and both wing lengths were measured as the distance from the axillary incision to the apical margin excluding the fringe [28].

### Statistical Analysis

As a test for whether DENV-2 altered any of the five tested components of *Ae. aegypti* blood feeding behavior, we used survival analysis to censor the time at which mosquitoes started feeding, probing and ingesting blood as the censoring event. We had a total of four curves, which were derived from the two populations (field/lab) and one treatment (infected/control mosquitoes). Initially, we performed a Log-Rank test to compare the four curves. If this first test revealed statistically significant differences between the four curves, we then ran a paired Log-Rank with Bonferroni correction to identify which curves differed from the others. Finally, the number of probes between control and infected groups in weeks 1 to 3 after DENV infection were compared using a Mann-Whitney *U* test.

*Aedes aegypti* longevity (which was defined by the day each mosquito died) was non-normally distributed, but the square-root of longevity satisfied the assumption of normality (Shapiro-Wilk *W* = 0.996, *P* = 0.081). We analyzed the effect of treatment (control or infected), mosquito population (field or lab) and looked for a correlation between wing length and mosquito longevity by using an ANOVA. We included in our initial model all main effects and interactions, and then deleted the highest order interaction (three way) when it was not significant, and retested the reduced model.

We performed a log-rank test to compare on a two-sample basis the survival distribution of *Ae. aegypti* females from control/treatments as well as field/lab populations. Herein, we define survival rate as the number of individuals still alive as a function of time. If log-rank detected that at least one of the four curves was statistically different, paired comparisons with Bonferroni corrections were performed.

Fecundity was analyzed by considering the first four clutches of eggs laid, as only a small number of females laid eggs when they were more than five weeks old, precluding adequate numbers for analysis. We analyzed two aspects of fecundity. First, we analyzed the oviposition success, i.e. likelihood that a mosquito laid at least one egg (at a given clutch) with a logistic analysis that included treatment, population, wing length and clutch-number (i.e. age), again backwards-eliminating the higher order interactions if they were non-significant. Second, we analyzed the number of eggs per clutch from those mosquitoes that laid at least one egg, using a repeated measures analysis. We square-root transformed the number of eggs to satisfy the assumptions of normality. We included clutch-number as the variable repeatedly measured and estimated the effects of treatment, population and looked for a correlation to wing length. All analyses were carried out with the statistical software JMP 9 (http://www.jmp.com/).

### Ethical Issues

The use of anesthetized mice to blood feed mosquitoes was authorized by Fiocruz Ethical Committee for Animal Use (CEUA L-0007/09).

## Results

### Vector Competence Assay

From a total of 49 mosquitoes that received an infective blood meal, 45 of them survived up to 14 dpi. Of these, 42 (93.3%) had DENV in the head, suggesting a satisfactory susceptibility of our mosquitoes to the DENV-2 strain used.

### Effect of DENV-2 on *Ae. aegypti* Life-history Traits

We used a total of 173 *Ae. aegypti* females in this study, 123 mosquitoes were infected with DENV-2 and 50 were controls, i.e. received only uninfected blood meals. Of the 173 mosquitoes, 96 (55%) were from the field population and 77 (45%) were from the Paea strain.

### Feeding Behavior

Overall, DENV-2 produced some age-dependent alterations on *Aedes aegypti* feeding behavior. Host-seeking behavior, i.e. efficiency of locating the mouse and beginning the blood feeding process was not influenced by DENV-2 infection at any of the post-infection time points evaluated (Table 1). Probing time was also unaffected by DENV-2 infection, indicating that infected individuals do not to start blood-feeding faster, as originally hypothesized. However, although the ingestion time for infected and control individuals was statistically similar in the 1^st^ week, in the 2^nd^ and 3^rd^ weeks infected mosquitoes spent more time ingesting blood compared to uninfected counterparts (Second week: χ^2^ = 8.38, df = 3, *P* = 0.031; Third week: χ^2^ = 10.25, df = 3, *P* = 0.017). Finally, on the third week after infection, the overall feeding time of DENV-2 infected females was longer than that observed for controls (Table 1).

**Table 1:** Survival analysis of the feeding behavior of *Aedes aegypti* females in the three weeks following DENV-2 infection.

	First week (7 dpi)			Second week (14 dpi)			Third week (21 dpi)		
	df	Chi	P	df	Chi	P	df	Chi	P
Start time	3	6.41	0.093	3	4.75	0.19	3	5.61	0.13
Probing time	3	7.51	0.057	3	4.65	0.2	3	3.01	0.39
Ingestion time	3	2.77	0.43	3	8.38	**0.031**	3	10.25	**0.017**
Feeding time	3	1.29	0.73	3	1.13	0.77	3	8.29	**0.04**

Values in bold represent statistically significant differences between infected and non-infected mosquitoes.

The average number of probes per female was not significantly different between infected and uninfected mosquitoes (First week: *U* = 1323.5, *P* = 0.657; Second week: *U* = 923, *P* = 0.305; Third week: *U* = 640.5, *P* = 0.719).

### Survival

**Figure 1:**
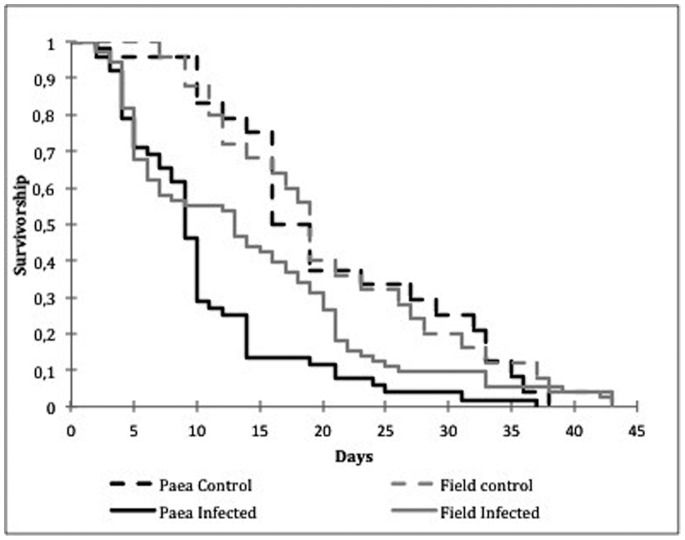
Survival curves of *Aedes aegypti* females from a field population and the laboratory Paea strain, infected with DENV-2 or controls (non-infected).

Overall, for both mosquito populations combined, uninfected *Ae. aegypti* females lived longer (median = 18 days) than the DENV-infected group (median = 11 days). Infected mosquitoes exhibited higher mortality than controls (χ^2^ = 22.7, *P*<0.001), especially on the first ten days after DENV-2 infection (Figure 1). The two mosquito populations reacted differently after being challenged with DENV-2. *Ae. aegypti* females from Paea strain had shorter survival when infected (χ^2^ = 16.5, *P*<0.001), whereas DENV-2 infection had no influence on the survivorship of mosquitoes from the field population (χ^2^ = 3.4, *P* = 0.063). When uninfected, mosquitoes from the Paea strain had a similar survival rate to that observed for field population females. When infected, the Paea mosquitoes survived less than the field population (χ^2^ = 5.44, P = 0.02). When considering one single point on the mortality curve we observed that 75.2% and 34.3% of the mosquitoes from infected and control groups, respectively, were still alive on the 14^th^ day after infection, when the virus can be expected to have completed its extrinsic incubation period in the constant temperature of 27±2°C of our insectary.

DENV-infected females demonstrated significantly higher mortality than controls (Table 2). Remarkably, mosquito wing size and population, as well as the interactions among tested variables were not statistically informative in the determination of mosquito mortality (Table 2).

**Table 2:** Analysis of variance of the square-root of survival of *Aedes aegypti* females.

Source	d.f.	Sum of squares	F	P – value
Population	1	0.004	0.049	0.825
Treatment	1	1.769	20.75	**<0.001**
Wing size	1	0.142	1.671	0.198
Population ANDTreatment	1	0.001	0.001	0.979
Population ANDWing size	1	0.141	1.659	0.201
Treatment ANDWing size	1	0.177	2.082	0.151
Error	126	10,74		

### Fecundity

**Figure 2:**
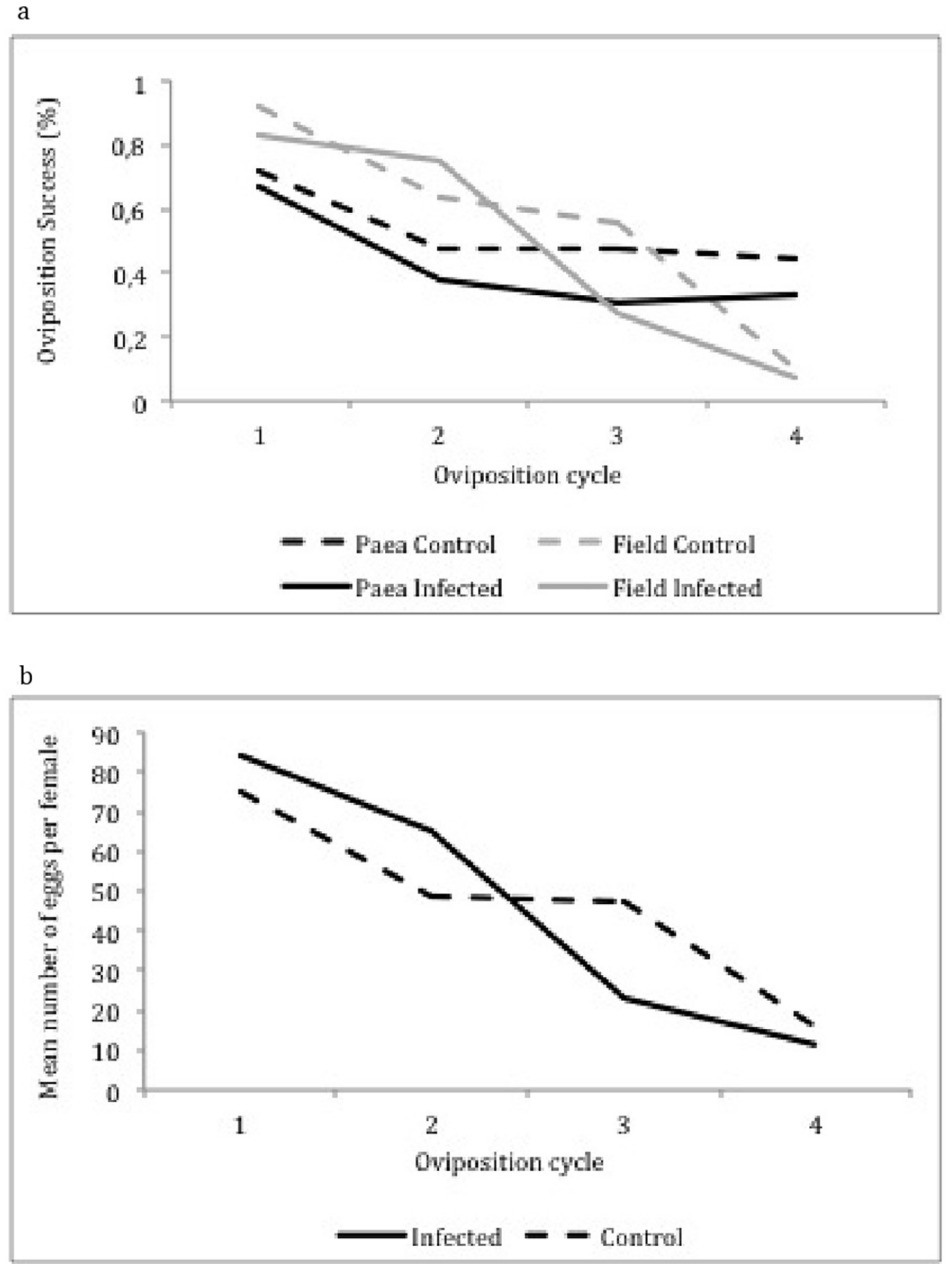
Aspects of *Aedes aegypti* fecundity. (A) Effect of DENV infection on the oviposition success of *Ae. aegypti* females during four oviposition cycles and (B) the mean number of eggs laid per female per oviposition cycle.

The first part of our analysis of fecundity considered whether or not a female laid eggs at all. Similar proportions, 86% of infected and 78.8% of control *Ae. aegypti* females laid at least one egg during any of the four oviposition cycles evaluated here. Overall, egg-laying success was strongly affected by mosquito age, (χ^2^ = 15.03, d.f. = 3, *P* = 0.0016) decreasing with female age from 78.04% success rate at the first clutch to 70.84% at the fourth egg clutch (Table 3). There was a significant interaction between mosquito age and treatment, with oviposition success dropping more rapidly with age in infected mosquitoes, especially for the third and fourth egg clutches (Figure 2A). Another important interaction was observed between mosquito age and population, with egg-laying success dropping faster with age in mosquitoes from the field population rather that in those from the Paea strain.

**Table 3:** Logistic regression analysis of the influence of mosquito age when they lay eggs, population, treatment and wing size on the success of oviposition.

Source	d.f.	?^2^	*P-value*
Age	3	15.305	0.0016
Population	1	0.025	0.8733
Treatment	1	0.135	0.7125
Wing size	1	1.334	0.2480
Age AND Population	3	9.033	**0.0289**
Age AND Treatment	3	14.405	**0.0024**
Age AND Wing size	3	2.639	0.4506
Population AND Wing size	1	0.002	0.9591
Population AND Treatment	1	0.914	0.3389
Treatment AND Wing size	1	0.186	0.6659

The second part of the analysis considered the number of eggs from females that laid at least one egg (Table 4). Overall, we observed a tendency of a decreasing number of eggs with time irrespective of mosquito treatment and population, from a mean of 104.78 in the first clutch to 86.57 in the fourth clutch (Figure 2B). The number of eggs laid by infected females dropped more rapidly than it did in the control group after the third oviposition cycle and irrespective of mosquito population (Figure 2B, Table 4).

**Table 4:** Repeated measures analysis (with clutch size as the repeatedly measured variable) of the square-root of the number of eggs laid by *Aedes aegypti* females.

Source	Numerator d.f.	Denominator d.f	*F*	*P*
Clutch AND Treatment	2	11	6.427	0.014
Clutch AND Population	2	11	0.928	0.424
Clutch AND Wing size	2	11	1.631	0.239

## Discussion

We designed experiments to study the potential impact of DENV infection on some *Ae. aegypti* life-history traits by orally challenging female mosquitoes. We showed that *Ae. aegypti* females presented significant negative effects in longevity, fecundity and oviposition success once infected with DENV-2. Furthermore, DENV-infected mosquitoes required more time to ingest blood and complete a blood meal. This phenomenon observed two and three weeks after infection, when mosquitoes were around 14 and 20 days old. By using the F1 generation of a field-collected *Ae. aegypti* population that was orally infected rather than intrathoracically challenged and a dengue strain with low passage history we created a close simulation of natural transmission.

The potential effect of DENV infection on *Ae. aegypti* feeding behavior has received minimal attention, with only a few studies addressing this subject despite its epidemiological relevance. Two studies considered the biting rate of DENV-infected *Ae. aegypti*, but presented opposing conclusions. Using mosquitoes from long-established laboratory colonies, a DENV-2 with a long but unknown passage history and infecting mosquitoes by intra-thoracic inoculation, (i.e., a non-natural system) Putnam and Scott [20] found no evidence that DENV-2 influences the mosquito’s feeding behavior (i.e., biting rate). Using F1 to F3 generations of field-collected *Ae. aegypti* intra-thoracically inoculated with a low passage DENV-3 strain, it was demonstrated that the time required for blood-feeding and the time spent during probing was longer in dengue-infected mosquitoes than in uninfected individuals [13].

Our results showed age-dependent alterations in the feeding behavior of infected individuals in comparison with their uninfected counterparts, especially by increasing the time required to ingest blood to repletion and on the total feeding time. If a mosquito takes longer to complete a blood meal due to DENV infection, the chances of being killed by host defensive behavior are increased, thus potentially reducing parasite transmission rate would not be maximized [29]. The finding of age-dependent modifications might be one of the potential outcomes of DENV infection considering the dynamics and tropism of DENV in the *Ae. aegypti* body, which was elegantly shown elsewhere [30]. At 7 dpi, less that 50% of mosquitoes from F3 to F6 generation of the Chetumal strain had DENV in their head tissues, and between 51 to 75% of them had DENV in their salivary glands. At 14 and 21 dpi, up to 95% of *Ae. aegypti* females had DENV in their head tissues and salivary glands [30]. Our data suggest that somehow the dissemination of DENV through the mosquito over time may be correlated with the age-dependent effects on the life-history traits we observed.

The effect of DENV infection on *Ae. aegypti* longevity is a crucial parameter to determine the vectorial capacity of a mosquito population. In order to support dengue transmission, a mosquito must survive at least 10–14 days. Despite its public health importance, little is known about the *Ae. aegypti*–DENV interactions, particularly the longevity of DENV-infected mosquitoes. One study reported that DENV-2 reduced mosquito longevity and survival rate of infected individuals [16]. These authors speculated that the high mortality of the infected group was due to an intense immune activation. While eliminating the viral infection, the immune response may create a biological burden that consequently increases mortality, as has been described for other vector-pathogen systems [16], [31]. The cellular and humoral immunity responses of *Ae. aegypti* mosquitoes to arboviruses such as DENV may use RNA intereference, a major component of the mosquito innate immune response, to modulate infection by producing molecules that inhibit virus replication [32]–[34]. Although there is no prior evidence that *Ae. aegypti* mosquitoes experience a fitness cost due to DENV-specific immune reactions, other vector-pathogen systems have reported similar findings [35]–[37].

The effects of DENV infection on *Ae. aegypti* were more evident on fecundity rather than on the feeding behavior and survival since infected mosquitoes were qualitatively (exhibited reduced oviposition success) and quantitatively (laid fewer eggs per female) affected. Joshi et al. [38] observed that the fertility and fecundity in vertically DENV-infected *Ae. aegypti* batches were lower than in control individuals. Our results considered horizontally (orally) infected mosquitoes and showed that the effect of DENV on fecundity varied over the mosquito lifespan. In the first two clutches, infected and uninfected mosquitoes laid similar numbers of eggs. On the third clutch, infected females had lower fecundity than the control treatment, corroborating the negative impact of DENV on the number of eggs laid per female per oviposition cycle [16]. *Ae. aegypti* females laid the eggs of their 2^nd^ and 3^rd^ clutches around 11 and 18 days after infection, respectively. It is possible that an immune response elicited by DENV infection created a fitness cost that was revealed by a reduction in fecundity. Furthermore, around 15 days after infection, several mosquito tissues such as the midgut, nervous system and salivary glands are severely infected by dengue virus [30]. At around 15 dpi the DENV may be found in females ovaries, which may have a direct impact on the oviposition success as well as the number of eggs laid per female per gonotrophic cycle [16], [30].

The biological relevance of the reduction of oviposition success and fecundity in DENV-infected individuals might be limited for several reasons. First, *Ae. aegypti* daily survival rates in the field suggest that only a small number of mosquitoes would survive to reach the 3^rd^ oviposition cycle, which is where we observed the strongest reduction in fecundity [39]. Additionally, because the incidence of DENV infection in natural mosquito population is extremely low, any differential effect on fecundity that DENV infection might impose on female mosquito is likely negligible. Parasite-induced fecundity reduction in other vector/parasite interactions has already been observed for the *Ae. aegypti*-DENV system [16], [40], as well as in *Leishmania*-infected sandflies [41], *Dirofilaria*-infected *Ae. trivittatus* mosquitoes [42] and *Onchocerca*-infected blackflies [43].

Overall, the outcome of infection and the potential effects of DENV on mosquito life-history traits must consider at least four interconnected points: (a) virus preparation (titer, serotype and passage history), (b) mosquito population, (c) mode of inoculation and (d) mosquito-virus genome interactions.

Infection of *Ae. aegypti* with DENV is closely correlated with the amount of virus ingested, and typically, requires as much as 10^6^ or more infectious units per orally administered milliliter to be infective [44], [45]. Likewise, mosquito survival and longevity also seem to be affected by virus titer, since higher mortality was observed with increasing viral RNA copies [16]. Previous studies examined different virus titers, however these studies used intrathoracic inoculation, a non-natural mode of transmission in which more viral particles are delivered directly to the insect hemocoele through bypassing the midgut infection and/or escape barriers [13], [17], [20]. Thus, it is possible that the increase observed in (i) the total time required for feeding [13] and (ii) the locomotor activity of DENV-infected females [17] was overestimated due to the mode of DENV transmission to mosquitoes.

Other important factors that can modify the outcome of a dengue infection are the origin and history of mosquito population as well as the passage history of virus strain. The infection rate for dengue virus increased from 52.0% in the F2 generation to 87.3% for the F20 generation of *Ae. aegypti formosus* collected in Africa [45]. Mosquitoes from the Paea strain had lower survival when infected with DENV-2 in comparison with the field population, a pattern opposite to what was previously observed [16]. Mosquito populations may interact differently with different DENV strains, with the outcome of infection being governed to a large extent by genotype-by-genotype (G×G) interactions between mosquito and virus in genetically diverse natural populations [46], [47]. The general definition of G×G interactions allows the characterization of phenotypes from macroscopic traits such as lifespan [48], [49]. Thus, it seems reasonable to speculate that our experimental design, which used (i) sympatric vector-virus populations, with (ii) mosquitoes recently collected from the field, (iii) DENV with low passage history provided (IV) offered to *Ae. aegypti* by artificial membrane feeders would be the most appropriate form of studying mosquito-virus interactions. Herein, we used a low-passage DENV to orally infect the F1 generation of field-collected mosquitoes, a system that best models and reflects natural transmission [50].

We have described significant new information regarding the negative impact of DENV-2 infection on *Ae. aegypti* biology. Overall, we observed that infected mosquitoes need more time to complete a blood meal, showed reduced survivorship, showed reduced egg laying incidence and reduced egg number. Potentially, the fitness cost of DENV infection observed herein might be of limited relevance to natural mosquito population dynamics by at least three reasons. First, the observed reduction on mosquito survivorship, oviposition success and fecundity was highly associated with aging, i.e. mosquitoes must survive at least around 20 days. Before that, fitness cost of DENV infection looks less intense. Second, *Ae. aegypti* mosquitoes has limited daily survival. For instance, mark-release-recapture experiments estimated the probability of daily survival of *Ae. aegypti* females as around 0.80–0.85 [39]. Therefore, the number of mosquitoes that would live up to the day when the fitness cost is more intensive would be small. Finally, the number of naturally DENV-infected mosquitoes on natural populations is extremely low. Thus, overall, the cost of DENV infection may have limited influence on natural mosquito population dynamic.
